# Annotations

**Published:** 1904-07-30

**Authors:** 


### Annotations.

### Society for the Study of Disease in Children.

The annual report of this Society, which was submitted to a general meeting of the members on July 15, announces a highly satisfactory state of affairs. Though only founded in 1900, the Society can now boast over 300 members, and an unusually large proportion of these regularly attend the meetings. The annual volume of "Transactions" is a most valuable record of clinical and pathological work, and from it one can readily see how attractive is the material presented at the Society's meetings. The financial position is not less satisfactory. Not only are current expenses successfully met, but there is an accumulated balance of over £200, in addition to an endowed lectureship of £20 per annum. All this may be taken as testimony that the Society meets a real want and that it is conducted with energy and good judgment. The only criticism we have heard passed upon the policy of the Society is that it does not adequately appreciate the significance and value of its annual general meeting. This appears to be invariably held either at the private residence of one of the officials or in association with a social dinner. No doubt most of the business to be transacted is of a formal character and excites but little interest. All the same, it is much to be desired that the one opportunity possessed by the non-official members of expressing their views of the Society's affairs should not be unfairly invaded. Every governing body is the better for criticism, and the council of a medical society is no exception to this rule. To tie up the one general meeting of the year to a social function is practically to destroy it as an opportunity for free speech on the part of the private member.

### Voyages d'Études Médicales.

At the request of some of our French *confrères* we have the pleasure of drawing the attention of our readers to the plan and scheme of the Voyages d'Études Médicales. The "voyages," in brief, are organised visits by parties of medical men to the health resorts, mineral springs, sanatoriums, and climatic stations of France, and are open to members of the profession of all nationalities. For purposes of the scheme the country has been divided into appropriate districts, and one of these is selected for inspection in each year. On the present occasion the tour is to be to the central district and Auvergne, and will include visits to such important places as Évaux, La Bourboule, Mont Dore, Royat, Châtel Guyon, Vichy, Saint Honoré-les-Bains, and others. The rendezvous is Lamotte-Beuvron on the morning of Saturday, September 3rd, and the tour will conclude on September 15th at Pougues. The details of the programme for each day are fully arranged, and anyone wishing to take part in the trip has but to send his name, and if he wishes, also, that of his wife, to Dr. Carron de la Carrière, 2 Rue Lincoln, Paris, not later than August 15th. The inclusive charge for the tour is 250 francs (£10), which covers travelling expenses, hotels, meals, *pourboires*, etc., between Lamotte-Beuvron and Pougues. Further, a reduction of half the fare may be obtained on the French railways both in travelling to and returning from the official tour. No better method of obtaining a direct personal knowledge of the French health resorts can possibly be found, and it is to be noted that Professor Landouzy will accompany the party and will give at each town visited an account of the therapeutic value of its climatic and balneary attractions. On the basis of a personal experience we are able to speak in appreciative terms of the educational success of these tours and of the social charm which attends them. The arrangements leave nothing to be desired, and visitors are received with so friendly and courteous a welcome that a sense of comradeship at once animates the entire company. As a charming and economical holiday there are few rivals to the scheme of the "V. E. M.," to use the familiar abbreviation current amongst the adherents of these tours.

### Tropical Diseases and Sanitation in India.

At the Health Congress, which was held at Folkestone this year and closed its sittings on Tuesday, the Tropical Medicine Section attracted a great deal of interest. The president of the section was Professor W. J. Simpson, M.D., of King's College, who gave an address on "Preventive Work in Tropical Medicine,", and dwelt at length on the question of sanitary organisation which, he said, was so much needed in the tropics, not only against malaria, but also against other diseases. Subsequently, by special request, Mr. James Cantlie, M.B, spoke on "beri-beri." He described in detail the geographical distribution and treatment of the disease, which he supplemented by giving its signs, symptoms, and causes. In the past, Mr. Cantlie affirmed, no attention had been paid to beri-beri, but at the present time a dozen cases could be produced in London. It was, he thought, peculiar how diseases became fashionable, and to-day beri-beri seemed to be fashionable. People who had money invested in South Africa were interesting themselves in it, and he rejoiced, for the sake of the Empire, that at last notice had been taken of one of its greatest scourges. Mr. Cantlie explained the different types of the disease, and mentioned places where it had largely existed, though under other names. We do not object to beri-beri being a fashionable disease, if the result is the mitigation of the malady; but mere chatter about its origin, its progress, and its character in West End drawing-rooms will not do much in that direction. We are glad to see that the proceedings of the Tropical Medical Section did not end with talk. A resolution was passed urging the Council of the Royal Institute of Public Health to approach the Secretary of State for India, and the Viceroy, for the purpose of laying before them the importance of a largely-increased special sanitary service for India. If this recommendation is acted upon, some practical result may be the outcome of their deliberations. As Lord Curzon is now in England, it would be easy for the Secretary of State to confer with him on the subject, and if a weighty representation is made of the needs of the case, it is not perhaps too much to hope that a service which notoriously needs strengthening will be augmented and rendered more efficient.

